# Characterization of fruit production and market performance in northwest Ethiopia

**DOI:** 10.1186/s43170-023-00149-3

**Published:** 2023-04-24

**Authors:** Mengistie Mossie, Alemseged Gerezgiher, Zemen Ayalew, Zerihun Nigussie, Asres Elias

**Affiliations:** 1 College of Agriculture and Environmental Sciences, Bahir Dar University, P.O. Box 79, Bahir Dar, Ethiopia; 2 Center for Rural Development Studies, Addis Ababa University, P.O. Box 1176, Addis Ababa, Ethiopia; 3 Faculty of Agriculture, Tottori University, 4-101 Koyama-Minami, Tottori, 680-8550 Japan

**Keywords:** Production, Post-harvest loss, Marketing margin, Fruits, Ethiopia

## Abstract

**Background:**

In Ethiopia, fruits pose a significant production and marketing challenge for farm households that significantly affect their farm profitability due to their perishability and unpredictable seasonal pricing. For instance, seasonally, market prices vary depending on the quality and quantity of fruit products available on the market. Stemming from this logical ground, this study is initiated with the objective of characterizing production systems and market performance of fruits in Ethiopia, focusing on apple and mango crops.

**Methods:**

A random sampling approach was used for producers and snowball sampling for traders when selecting survey participants. A pre-tested survey questionnaire was used for data collection. Descriptive statistics and market margins were used for statistical analysis.

**Results:**

Post-harvest wastage is preventing farmers from receiving anticipated revenue, implying that approximately 31.8 and 26.1% of the total mango and apple produce was lost, respectively. According to the survey results, there was no measurement consistency among farmers, local collectors, and small retailers. District level collectors received a higher margin share (42.66 and 40.18% of apple and mango, respectively) than other actors in the chain, which was unjustified given their contribution to the market chain. Farmers were comparatively hampered by the market since they earned the lowest share (33.34 and 15.08% of apple and mango, respectively) of consumer prices indicating that the apple and mango market chain performance is poor. As a consequence, these all deter farmers from producing in large quantities, quality, and also uncertainty (fair failure in the mind of farmers) in the marketing of apples and mangoes.

**Conclusions:**

The awareness of small-scale farmers about most of the agronomic practices including insect pests and diseases were very low. Hence, this study recommended that there is an urgent need from district agricultural offices to improve mango and apple production and marketing systems in the study districts.

## Background

In developing countries, the share of high-value agri-food products such as fruits and vegetables is rapidly increasing in consumers’ diets (Joosten et al. 2015; van Berkum 2021). Currently, the majority of these products are distributed through multilevel marketing schemes rather than being sent directly from producers to end customers (Barrett et al. 2019; Nájera 2017). Likewise, integrating rural households into lucrative agricultural markets is one of the most likely strategies to increase their livelihoods and food security (Orr et al. 2018). Again, this calls for system thinking to overcome obstacles that prevent farmers from participating in profitable local and international marketplaces (Tschirley et al. 2015; Lundy et al. 2012). In this case, agricultural market chains that link farm households with traders and consumers of agricultural products are of particular interest (Lie 2017).

Typically, Sub-Saharan African farmers do not have adequate access to market information (Quisumbing et al. 2015; Barrett et al. 2022). For instance, farmers might not comprehend the worth of their farm products (FAO 2019; Bokelmann and Adamseged 2016). Ethiopia is no exception to this truth. For exports of agricultural products like coffee, hides and skin, dairy, and sesame, market chain analysis was mostly carried out in Ethiopia (Bereda et al. 2016). As a result, market chain analysis is essential for satisfying demand by improving the level of competition and increasing the productivity of products like fruits. In various regions of Ethiopia, some empirical study on the fruit market chain is being done, however it has some limitations. Firstly, since these studies are primarily focused on Central and Southern Ethiopia (e.g., Tarekegn et al. 2020; Gebre et al. 2020; Honja 2014; Getahun et al. 2018; Mengesha et al. 2019), the contextual relevance (e.g., institutional and infrastructure) to north-western part of Ethiopia may be limited.

**Fig. 1:**
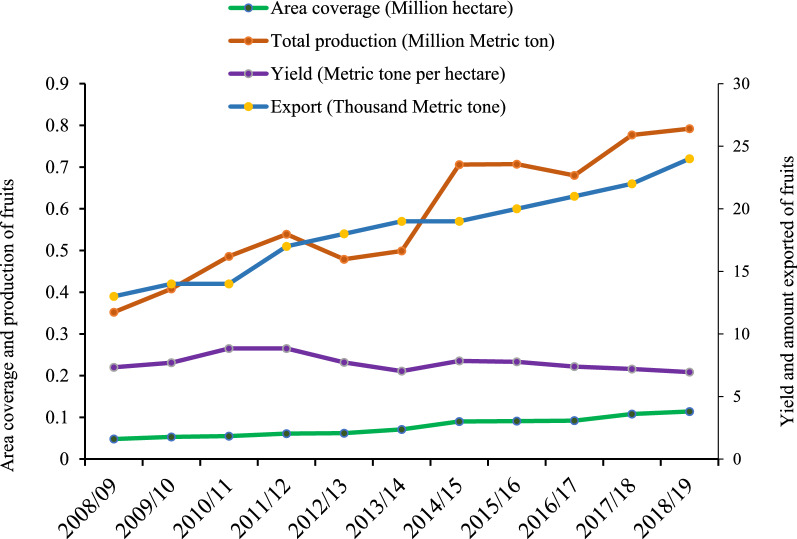
Land area and production trends in Ethiopia’s key fruit crops. Source Central Statistical Agency of Ethiopia (CSA 2019)

Because fruits are perishable and their prices change seasonally, farmers encounter various production and marketing challenges that have a significant impact on their farm profitability (Tschirley et al. 2010). For instance, market prices fluctuate seasonally based on the variety and quality of fruit products available. Prices often change dramatically, even within a single day, particularly in the wholesale and retail markets (Woolfrey et al. 2021). The methods used in fruit production and the market chain are therefore understudied and need more research. Hence, using data from participants in the fruit market chain, this study analyses production systems and quantifies the benefit distribution across participants in the Ethiopian apple and mango market chain. For instance, fruit farming contributes significantly to Ethiopia's economy and supports about five million rural farmers (GAIN 2018). In Ethiopia, fruit cultivation occupied over 114,421.81 hectares during the 2018/19 cropping season, producing a total of 7,924,306.92 quintals. Figure 1 depicts the cultivated area coverage, production volume, and yield of major fruits from 2008/2009 to 2018/2019, including strawberries, apples, bananas, papayas, avocados, mangoes, and pineapples. Moreover, inadequate harvesting and management, disease and insect infestations, erratic weather patterns, and all of these are linked to the observed fluctuations (CSA 2019).

Mango and apple fruits are categorized as the most commercially significant crops grown and frequently consumed worldwide (Solís-Fuentes and del Carmen Durán-de-Bazúa 2011). Mango (*Mangifera-indica*) is regarded as “the king of fruits”, making the crop valuable for ensuring food security, especially in emerging nations where this issue is still present (Ullah et al. 2010). Production of mangoes grew globally, going from 24.70 million metric tons in 2005 to 54.80 million metric tons in 2018/2019 (FAOStat. 2020). Ethiopia's mango production increased by 45% between 2014 and 2018, from 70,000 metric tons to 105,000 metric tons (GAIN 2018). Mangoes are exported on both a local and global scale. The main market for Ethiopian mangoes is Djibouti. Mango fruit processing for the purpose of preservation and value addition is uncommon in the study areas. Mango processing, on the other hand, is handled by juice houses, cafés, restaurants and hotels. Some of the most important biotic problems limiting mango yield are insect pests. White mango scale is one of the most common insect pests of mango plants in Ethiopia, along with the study locations. In the meantime, pesticides to control white mango scale were developed in Ethiopia, including Folmit 500 SL, Methidathion 400 EC, and Movento 150 OD (Ayalew et al. 2015; Ofgaa and Emana 2015).

Apple (*Malus domestica*) production accounts for more than half of the world’s deciduous fruit tree farming. In 2018/2019, about 86 million tons of apple was produced worldwide (FAOStat. 2020). Aside from their flavor and nutritional value, apples are also prized for being easily cleaned and preferably peeled, which is important given the ongoing COVID-19 pandemic (WHO 2020): “*An apple a day keeps the doctor away*”. Many highlands in Ethiopia, particularly the Upper-Blue Nile, have seen an increase in apple fruit production (Tamirat and Muluken 2018; Mossie et al. 2021a). The main varieties planted in the country are Kent, Apple Mango, Tommy Atkins, and Keitt (Bekele et al. 2020). There are 818 hectares of apple farms in the Amhara region, and 45,000 quintals of apples were harvested there during the 2019 growing season (BoA 2020). In the same year, Ethiopia imports 1,659 metric tons of fresh apple mainly from France and South Africa, 40% and 24%, respectively. In Ethiopia, apple fruit is often consumed in the form of fresh fruits. As a result, there are no industrial processed apple fruits consumed locally. In spite of this, consumption of fresh juices and processed apple juice products imported from the Gulf region is growing in major cities and other urban areas (CSA 2019).

## Methods

### Study area description

**Fig. 2:**
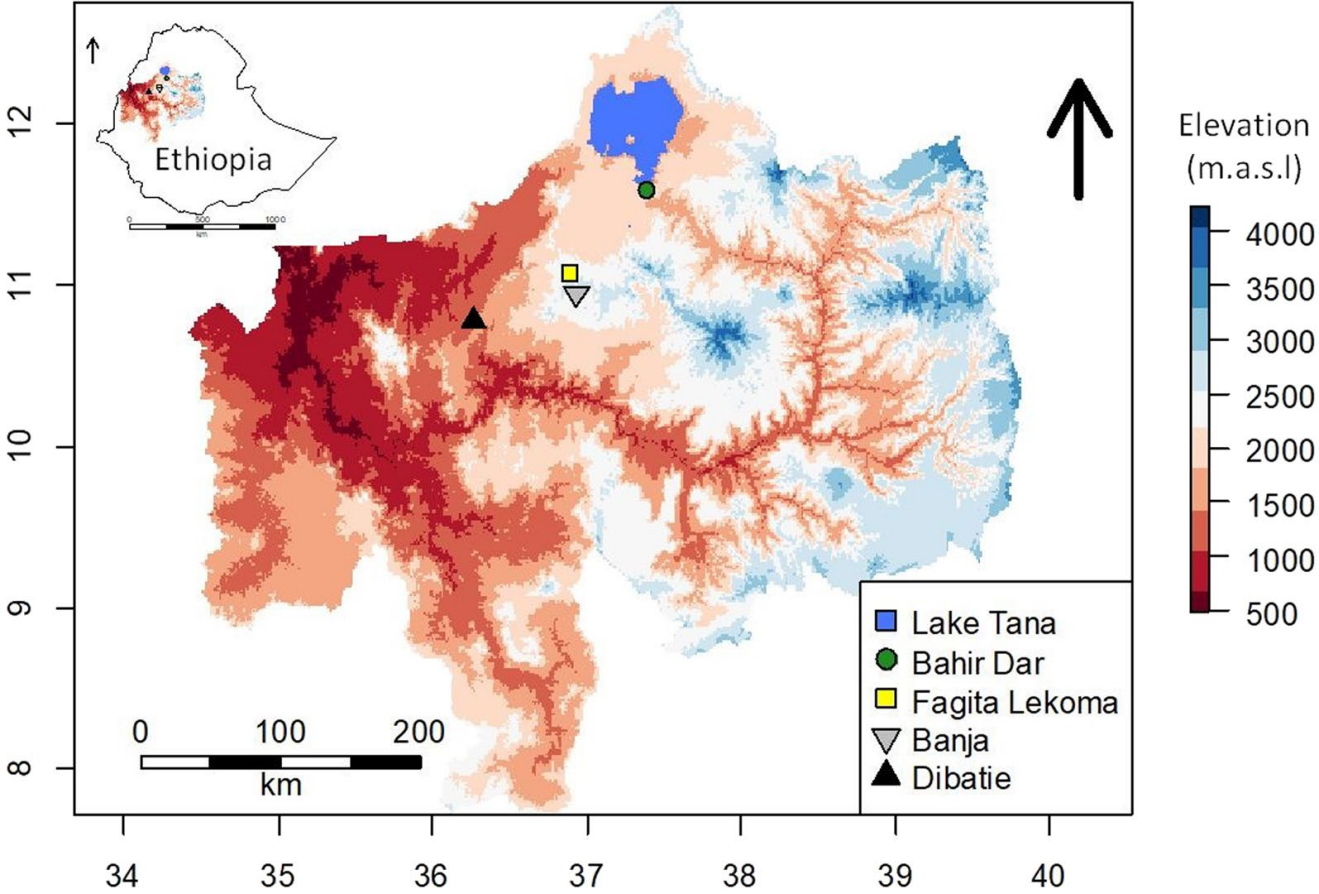
Location map of the study areas

The present study was carried out in the Banja, Dibatie, Fagita Lekoma, and Bahir Dar Zuria districts (Fig. 2). These districts are located in northwestern highlands of the country, and the area is the source of around 86% of the Nile River's water (Block 2006). In terms of study area selection, the researchers’ experience of the issue under study, and their affiliation with the survey area, were important. The main sources of income for the households in the study areas are animal husbandry and rain-fed mixed subsistence crop cultivation (Nigussie et al. 2017; Mossie et al. 2021b). Fruit crops like mango and apple are important agricultural contributors as well, making them a priority focus for development in Ethiopia’s northwestern highlands. Table 1 describes the biophysical characteristics of the study districts as high, mid, and low elevations in order of elevation.

**Table 1:** Description of the study areas. Source Profiles of each district's socioeconomic situation (2019)

Features (unit)	Study districts			
	Dibatie	Fagita Lekoma	Bahir Dar Zuria	Banja
Temperature (℃)	25–32	9–25	15–28	9–26
Annual rainfall (mm)	850–1200	1951–3424	895–2037	1958–3465
Altitude (m a.s.l.)	1479–1709	1800–2900	1922–2250	1850–2925
Agro-ecological zone	Tropical hot humid	Moist subtropical	Humid subtropical	Moist subtropical
Dominant livestock	Cattle, goats, and donkeys	Cattle, horses, and sheep	Cattle, goats, sheep, and donkeys	Cattle, horses, and sheep
Dominant cash crops	Mango, groundnut and coffee	Apple, Potatoes, and garlic,	Khat, mango, papaya, avocado	Potatoes, apple, and garlic,
Dominant staple crops	Maize and millet	Barley and teff	Millet, teff, wheat, maize	Teff and barley

### Sampling procedures

A standardized survey questionnaire was used to interview study participants (i.e., household heads) in four districts. A multi-stage sampling technique was used to sample farm household heads, which included both purposive and random sampling methods. Firstly, four districts (i.e., Bahir Dar Zuria and Dibatie from the mango producing districts; Fagita Lekoma and Banja from the apple-producing districts) were purposively chosen. These districts were selected in order to capture socioeconomic situations, agro-climate zone differences, and fruit producing experiences. Secondly, ten sample kebeles were randomly selected. Finally, utilizing the Mugenda and Mugenda (2003) table, a sample of 384 survey households was proportionally picked.

On the other hand, for this study, the sites for the trader surveys were market towns in which a good sample of fruit traders existed. Accordingly, based on the flow of fruits, three markets (*Chagni, Dibatie, Bahir Dar and Enjibara*) were selected as the main apple and mango marketing sites for the study areas. Due to the lack of a recorded list of the population of traders in this situation and the traders' opportunistic behavior, sampling is a particularly challenging task. Therefore, a snowball sampling technique (Magigi 2015) was utilized to interview traders (collectors, wholesalers and retailers) from designated markets. The consumers’ survey was taken from the customers of major retail shops and wholesalers (of ETFRUIT shops) from specified towns by distributing questionnaires at the time of purchase.

### Data analysis approaches

As fruit products pass sequentially through the different levels, transactions occur among key participants in the market chain. Marketing margin, which is composed of expenses and profits, is indeed a useful method to assess the performance of a marketing system (Nzima et al. 2014). The cost and pricing information gathered from the respondents was used to calculate the gross marketing margin. The methods employed in this study to analyze the effectiveness of the apple and mango market chain were marketing margin and channel comparison. A marketing channel is a market structure composed of interdependent entities that connects the producer of the good with the consumer to convey the good to the consumer who will use it (Kotler and Armstrong 2004). The comparison of the channels is based on the volume of mango and apple produce that travelled through each network. Using the formula shown below, the entire marketing margin was estimated (Mendoza 1995).

1 $$Total\,Gross\,Marketing \,Margin = \left( {\frac{Consumer \,price - Producer \,price}{{Consumer \,price}}} \right)100$$

The producer's share in the consumer price, also known as the producers' gross margin, is the amount of the price that the consumer pays which belongs to the producer.

2 $$Producers\, Gross\, Margin \left( {GMM_{p} } \right)\left( {\frac{Consumer\Pr ice - Total \,Gross \,Marketing\, Margin}{{Consumer\Pr ice}}} \right)100$$

3 $$\left( {NMM} \right) = \left( {\frac{Total \,Gross\, Marketing\, Margin - Marketing \,cost}{{Consumer\,Price}}} \right)100$$

The formula illustrates how the producer share decreases as the marketing margin (NMM) increases and vice versa. It also provides a measure of how benefits are allocated among the producers and marketers.

## Result and discussions

### Distribution of survey participants based on production and marketing practices

Growing fruit crops like apple and mango under a variety of agro-climatic conditions can be very profitable and competitive, as well as offer opportunities in the current study. Unfortunately, small-scale mango and apple growers have not taken advantage of these prospects due to poor market pricing for their product and the high expense of post-harvest losses. For instance, post-harvest wastage inhibits farmers from earning the money they anticipated and shows that 31.8 percent of the entire mango production was lost. According to empirical studies, post-harvest losses in fruit are estimated to be 20–40% in underdeveloped nations and 5–20% in developed nations (Mashav 2010). Most people are unaware of how much food is wasted in market chain operations (i.e., from harvesting to consumption). For example, wind, birds, severe injury, and maturation stage are the main reasons why mango products are lost in Ethiopia (Hussen and Yimer 2013).

**Fig. 3:**
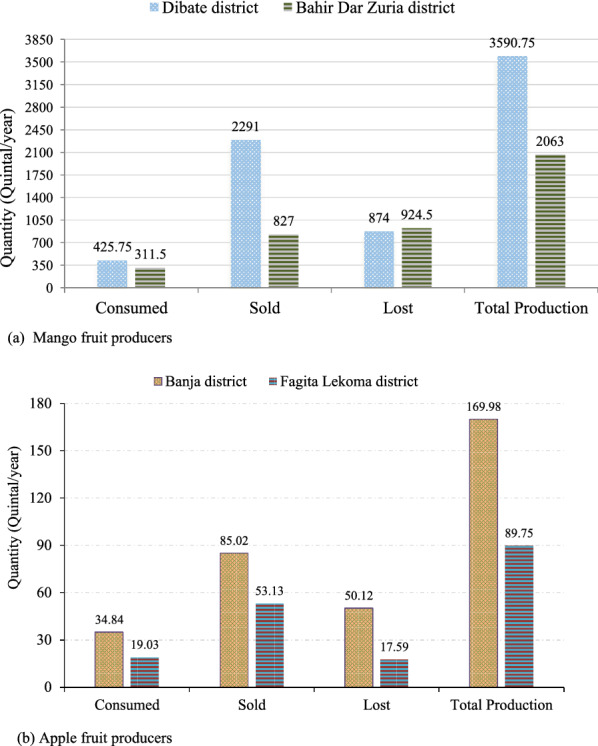
Households based on their total production, consumption, post-harvest loss and marketing practices. **a** Mango fruit producers. **b** Apple fruit producers. Source Own survey data (2020)

According to the descriptive result, depicted in Fig. 3, there are a sizable number of mango and apple growers in the research areas, each with varying levels of production and marketing intensity. In both mango producing areas (Bahir Dar Zuria and Dibatie), a substantial amount of the product is transported to market. This reinforces the notion that cash crops, such as mango, are planted largely for the market. This paper also shows that Dibatie district had a higher proportion of overall mango production than Bahir Dar Zuria. Approximately 13% of the mango produce was consumed at home, according to the findings. Birds, insects, diseases, physical injuries, and inadequate road transport and loading all contributed to significant fruit loss. This observation is consistent with the findings of Fetena and Lemma (2014), who identified on the challenge of disease and associated losses, as well as the study results of Honja (2014), which conducted a review of the mango market chain in Ethiopia.

Furthermore, there are farmers in apple-growing districts that have high levels of productivity. The Banja district's total productivity exceeds that of Fagita Lekoma. This could be due to the right use of common farming techniques including fencing, thinning and pruning, composting, manure, pest control, and others. Apple producers are also being hampered by post-harvest wastage, with estimates indicating that 26.1 percent of total apple production was lost (Fig. 3b). Overall, there is a significant difference in overall production, consumption, selling, and loss of apple produce based on district.

### Agronomic systems

Table 2 depicts some of the most common agronomic techniques used by mango and apple farmers in the research districts. According to the information gathered from the respondents, mango and apple trees are intercropped with other crops such as maize, coffee, peanuts, root crops, khat, legumes, and vegetable crops and are planted haphazardly without correct spacing. There is no cost that is directly associated with mango production because the crop husbandry practices such as land preparation, pruning and weeding, are indirectly done during the cultivation of other targeted annual crops. More than half of respondents in all study districts advocate intercropping their mango and apple with other crops. This result is supported by Dapaah et al. (2003), who disclosed that intercropping, as opposed to monocropping, is a popular method used over the world because it reduces crop failure risks, enhances land use efficiency, reduces soil erosion, and boosts yield stability. Disease and insect pest problems were reported by 40.90 percent and 34.10 percent of respondents, respectively, in their mango and apple orchards. Nevertheless, the majority of respondents (60.54 percent) stated that they did not apply any disease or insect pest management methods in their farming. Only 2.84 percent of all interviewees sprayed pesticide chemicals, meanwhile. In the research areas, white mango scale is one of the most prevalent insect pests of mango trees. Folmit 500 SL and Methidathion 400 EC were the types of pesticides that mango growers applied to reduce white scale disease infestation.

**Table 2:** Characteristics of apple and mango production in the study areas. Source: Own survey data (2020)

Items (%)	Bahir dar zuria	Districts			
		Fagita lekoma	Banja	Dibatie	Total
(1) Cropping systems practiced					
Intercropping	93.56	66.69	73.00	84.30	79.31
Mono-cropping	6.44	33.31	27.00	15.70	20.69
(2) Reasons for using intercropping					
To use the farmland efficiently	57.75	52.20	37.10	56.07	50.78
To increase soil fertility	22.40	4.41	25.40	21.10	18.33
Protects from disease and pests	1.31	3.26	0.60	2.22	1.85
For shading purpose	12.10	6.82	9.90	4.91	8.43
(3) Diseases and insect management methods adopted					
Removing dead trees	2.16	4.22	0.00	1.20	1.89
Weeding and hoeing	2.93	14.60	7.90	3.64	7.27
Spraying pesticide chemicals	5.00	2.80	1.11	2.43	2.84
Intercropping	14.30	12.62	13.93	12.00	13.21
Cultural methods	6.44	5.54	5.61	4.84	5.61
All of the above methods applied	16.41	6.90	4.04	7.22	8.64
No any controlling method used	52.76	53.32	67.41	68.67	60.54

According to field observations, the two most prevalent fungal diseases of mango in the research locations are “powdery mildew” and “anthracnose.” Apple scab, twig blight powdery mildew, and mildew are among the most common diseases that have reduced apple yield and productivity. Farmers spraying pesticide chemicals (e.g., endosulfan, diazinon). Insect pests that harm apple productivity include aphids, caterpillars, and scale borer.

### Distribution of respondents based on their fertilizer application

**Fig. 4:**
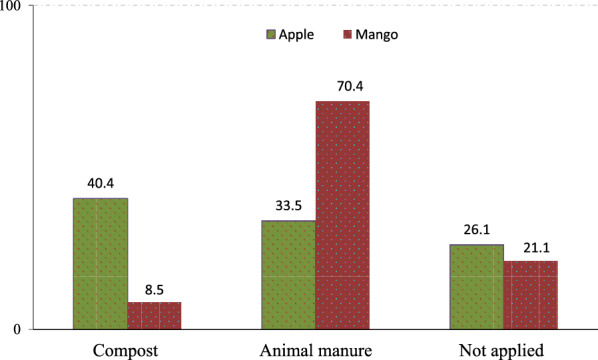
Percentage distribution of respondents based on their fertilizer application

In terms of fertilizer use, no apple or mango farmers used inorganic fertilizer (i.e., Urea and DAP) on fruit-growing land, whereas the majority of farmers used organic fertilizer (i.e., compost and manure). Figure 4 shows that the majority of the sampled apple growers (40.4 percent) used compost as a fertilizer, whereas 70.4 percent of mango growers used animal manure on their mango farms. As per the research results, 26.1 percent and 21.1 percent of apple and mango growers, respectively, did not use fertilizer on their farms. Alene (2017) highlighted that for highland fruit production, organic fertilizers such as compost and well-decomposed dung are preferable to chemical fertilizers. Compost is an organic matter that has been aerobically decomposed. Composting enhances soil biodiversity, which is fundamental for soil health (Kennedy 1999). Composting the soil improved its fertility, bulk density, water-holding capacity, and biological properties. Organic composts added to apple orchard soils have been shown to keep improving the flowering and growth of planting trees (Flavel and Murphy 2006; Reganold et al. 2001; Alalaf 2020).

### Seedling sources

**Fig. 5:**
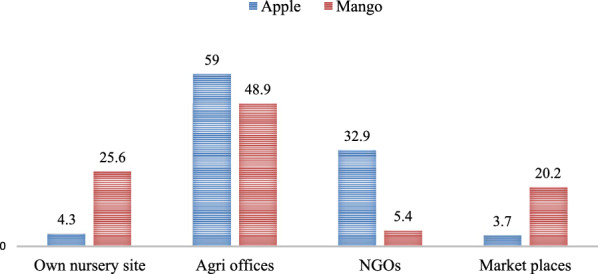
Sources of apple and mango seedlings (%)

The primary cultivars grown in the study area include Kent, and Tommy Atkins. According to the study, the district office of agriculture, non-governmental organizations (such as Japan International Cooperation Agency/JICA, Sustainable Land Management project, and Agri-service Ethiopia), and private seedling suppliers are currently the main seedling supply sources. According to Fig. 5, approximately 59% and 48.9% of respondents obtained apple and mango planting materials from district agricultural offices, respectively.

### Quantity measurement tools used during selling

In Ethiopia, the district-level marketplace is a well-known trading hub for a wide range of agricultural products. Nevertheless, the vast majority of economic agents exchange agricultural products in local markets using multiple, non-uniform units of measurement. The local units of measurement for fruits range from volumetric (basket, bowl) to weight measures (sack) and weight balance (kilogram) and counting. Overall, measurement system heterogeneity leads to significant measurement costs, market disintegration, and exchange inequitably (Abebe et al. 2018). The main cause of measurement problems at the transactional level stem from measurement error. The assumption of this viewpoint would be that measurement costs are caused by measuring instrument biases (Zhou et al. 2019).

**Fig. 6:**
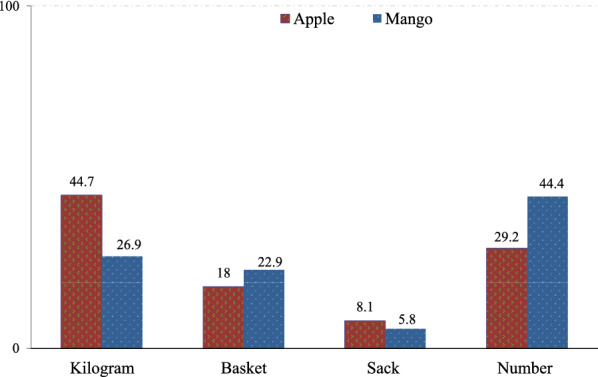
Quantity measurement tools used during selling (%)

Out of the total interviewed apple households, 44.7*%* used kilogram, 18*%* used basket, 8.1*%* used sack, and 29.2*%* used numbering (Fig. 6). About 26.9*%* of farmers measured mango sales in kilograms. Out of the total interviewed households, 22.9*%* used baskets, 5.8*%* used sacks, and 44.4*%* used numbering. According to the survey results, there was no measurement consistency among farmers, local collectors, and small retailers. In another case, farmers and local traders may lose a significant amount of money, particularly if the level of measurement costs is forecasted for the total number of transaction days made per year. In this way, the effects of a non-uniform system of measurement on the local economy are massive. Based on these realities, the study concluded that government initiatives and institutions are important for regulating the measurement actions of marketplace actors.

### Marketing system of apples and mangoes

According to the study, 32% of respondents sold their mango produce on the nearest local market roadsides, while 87% of households sold their apple produce on the farm field through collectors. The remainder were sold in the towns of Bahir Dar, Enjibara, and Chagni. Steps in the mango sell process include: “First, farmers told a rural collector to buy their produce. A collector returned to make other arrangements to locate and negotiate with a retail outlet and vendors. The buyer then proceeds to check the quality of mango product and negotiate prices. And then collectors assemble fruits from farmers in market places and farmlands using small trucks and pack animals in order to resell them to wholesalers or retailers. Their activities involve purchasing and assembling, sorting, and selling to wholesalers, who are usually transported on donkeys or carts to nearby towns like Chagni, Enjibara, or Bahir Dar. In the case of mango, they collect unripe mangos at the farm gate for 5–7 days before selling them at the assembly point to wholesalers from Enjibara and Bahir Dar. Apple collectors in the Enjibara area sell to Zengena Lake visitors and street vendors. On the other hand, retailers organize ‘collectors' groups to assemble mangoes from farmers and afterwards load them into vehicles that leave immediately for marketing. Wholesalers purchase mango products directly from farmers as well as collectors, usually in surplus areas, for resale to retailers and larger market centers with better financial capacity. They are actively focused in purchasing mango from farmers and collectors in greater quantities than any other actor and providing them to retailers as well as consumers. Retailers sell apples and mangoes, as well as many other fruit and vegetables like bananas and oranges. Their sales points are located in city markets, village centers, and along major roads.

### Marketing channel comparison

#### Apple marketing channel

The flow of products starts with the growers and finishes with the consumer. Marketing of apples in the study area begins at the local farms, traveling towards the storage area to the terminal markets. Output passes progressively through a variety of market players in such marketing chains, suggesting a sequence of ties in the market chain once it reaches the end-users. In fact, the number and form of market participants vary even between the final destinations of the commodities. The marketing participants were apple farmers, local collectors, street and shop sellers, and consumers. This study identified three major marketing networks (market channels) for apple production and moving to the different terminal markets. These are:

$${\text{Channel I}}:{\text{Growers}}\, \to \,{\text{Consumers }} = {9}.{7}\,{\text{Quintals}}\,({7}\% )$$

$${\text{Channel II}}:{\text{Growers}} \to \,{\text{Retailers}} \to {\text{Consumers }} = {16}.{5}\,{\text{Quintals }}({12}\% )$$

$${\text{Channel III}}:{\text{Growers}} \to {\text{Collectors}} \to {\text{Retailers}} \to {\text{Consumers}} = {112}\,{\text{Quintals }}({81}\% )$$

Due to the consistency and product quality aspect of the product, wholesalers are not willing to purchase apples. The marketing channel for apples is short because of this. A total of 138.15 quintals of apples were sold by farm respondents during the study year. About 81% of the overall volume sold by farmers passes via channel III (Farmers ⇒Collectors ⇒Retailers ⇒Consumers) with the largest share. This suggests that channel three is also more efficient at distributing large amounts of sales. This simply proves that a collector-created link was preferred in terms of absorbing a huge amount of apple products. The lowest apple quantity (7%) passes across channel I (Farmers ⇒Consumers). Unlike this result, (Tamirat and Muluken 2018) found that the greatest volume of apple fruit was marketed through channel II (Farmers ⇒Collectors ⇒Retailers ⇒Consumers).

#### Mango market channel

It was reported that 3118 quintals of mango were delivered to the marketplace by small-scale growers in the study year. This study identified seven major marketing networks (market channels) for mango production and moving to the different terminal markets. These are:

$${\text{Channel I}}: {\text{Growers}} \to {\text{Consumers }} = {174.61}{\text{ Quintals }}({5.6}\% )$$

$${\text{Channel II}}:{\text{Growers}} \to {\text{Retailers}} \to {\text{Consumers }} = {1016.92}\,{\text{Quintals }}({32.61}\% )$$

$${\text{Channel III}}:{\text{Growers}} \to {\text{Retailers}} \to \Pr {\text{ocessors}} \to {\text{Consumers}} = {162.6}0\,{\text{Quintals }}({5.21}\% )$$

$${\text{Channel IV}}:{\text{ Growers}} \to {\text{Collectors}} \to {\text{Wholesalers}} \to {\text{Consumers}} = { 52.50}{\text{ Quintals }}({1.70}\% )$$

$${\text{Channel V}}:{\text{Growers}} \to {\text{Collectors}} \to {\text{Wholesalers}} \to {\text{Processors}} \to {\text{Consumers}} = { 103.40}{\text{ Quintals }}({3.3}\% )$$

$${\text{Channel VI}}:{\text{Growers}} \to {\text{Collectors}} \to {\text{Wholesalers}} \to {\text{Retailers}} \to {\text{Consumers}} = {\text{1576 Quintals }}({50.55}\% )$$

$${\text{Channel VII}}:{\text{Growers}} \to {\text{Collectors}} \to {\text{Wholesalers}} \to {\text{Retailers}} \to {\text{Processors}} \to {\text{Consumers}} = 32.00{\text{Quintals }}({1.03}\% )$$

The lowest mango quantity (1.03%) passes across channel seven (Growers ⇒ Collectors ⇒Wholesalers ⇒ Retailers ⇒ Processors ⇒Consumers). About 50.55% of the overall volume sold by farmers passes via channel six with the largest share. This implies that channel 6 is more effective in terms of distributing large amount of mango sales. Kassa et al. (2017) identified the same result: the avocado and banana market chain, channel IV, which connected farmers to wholesalers via local collectors, was more effective in terms of supplying large volumes to terminal markets.

#### Margin analysis

Parallel to channel surveys, margin analysis can be performed and helps to assess how pro-poor or superior a market chain is. The marketing margins for apples and mango were determined as followed by taking the estimated sales prices of the respective actors in the market chain (growers, collectors, wholesalers, and retailers). The findings in Tables 3 and 4 showed that the cultivation of apples and mango and local marketing is beneficial for all economic actors. But the distribution of benefits along the market chain is inequitable.

**Table 3:** Marketing margin along actors in the domestic apple market chain. Source: Own survey data computations (2020)

Items (ETB/kg)	Apple growers	Local collector	Retailer	Horizontal sum
Purchase price	–	20	38	95.00
Cost of production	8.00	–	–	8.00
Cost of marketing:				
Transportation	1.50	2.00	–	3.50
Loading/unloading	–	–	–	–
Sorting/grading costs	–	2.50	–	2.50
Spoilage/loss	2.50	3.50	3.50	10.00
Other costs	3.50	2.00	5.00	10.50
Total cost of marketing	7.50	9.00	8.50	26.50
Total cost	15.50	9.00	8.50	31.50
Selling price (revenue)	28	36	45	101.00
Marketing margin	12.50	16.00	9.00	37.50
(%) share of margin	33.34	42.66	24.00	100.00

**Table 4:** Marketing margin along actors in the mango market chain. Source: Own field survey data (2020)

Items (ETB/Qt)	Growers	Local collector	Wholesaler	Retailer	Processor	Horizontal sum
Purchase price	–	455	1600	2150	2400	6605.00
Cost of production	25	–	–	–	–	25.00
Cost of marketing:						
Transportation	4.50	10.00	18.00	5.00	4.50	42.50
Loading/unloading	–	3.00	4.00	1.50	5.00	13.50
Sorting/grading costs	–	–	5.00	–	5.00	10.00
Spoilage/loss	8.50	12.00	7.00	11.50	3.50	42.50
Cost of processing	–	–	–	–	160.00	160.00
ther costs	–	10.00	25.00	15.00	5.00	55.00
Total cost of marketing	13.00	35.00	59.00	33.00	183.00	323.00
Total cost	38.00	35.00	59.00	33.00	183.00	348.00
Selling price (Revenue)	455.00	1600.00	2150.00	2550.00	2900.00	9955.00
Marketing margin	430	1145	550.00	400.00	500	3525.00
(%) share of margin	15.08	40.18	19.30	13.22	16.53	100

ETB (Ethiopian Birr) is the Ethiopian currency, and during the survey period 1 USD was about 29 ETB. 1 Qt (quintal) = 100 kg

#### Apple margin analysis

The structure of selling prices, margins, and price shares in each of the chain divisions is presented in Table 3. The findings showed that local collectors take the lion’s share of the final price (42.66%), suggesting that the price spreads vary during the process which means that farmers ought to be connected to fruit markets such as central urban markets and supermarkets. For market chain participants, margin analysis showed that about 66.66% of the apple market chain’s gross marketing margin belongs to apple traders, and farmers receive approximately 33.34% of the gross marketing margin. In fact, the size of the gross margin, according to KIT (2008), shows the amount of expenses, labor, loss of competitive markets, and transparent information. Even so, the market for apples was monopolized by a small number of traders, and price information was also not transparent in the study areas. The results of marketing margin calculations for actors in the apple market chain in the study area revealed that local collectors got the highest margins. In general, district-level collectors received a higher margin share than other actors in the chain, which was unjustified given their contribution to the market chain. This disproportionate share of advantages is a result of the interaction of power amongst actors. This statement suggests that the apple market chain performance is poor. Collectors are more favored than growers in this inefficient market chain. According to KIT (2008), this is common in Africa during peak season, when there is a sufficient supply.

#### Mango margin analysis

With regard to mango, local collectors take the highest (40.18%) in terms of margin share, followed by wholesalers (19.30%). Margin analysis showed that about 84.92% of the chain’s gross marketing margin belongs to mango traders, while farmers receive approximately 15.08% of the gross marketing margin. That is, while farmers doing all the work of producing the mango crop and bearing the associated risks, took only 15.08% of the benefit share (Table 4). All market players were usually operating at a profitable pace, but growers were comparatively hampered by the market since they earned the lowest share of consumer prices. This disproportionate share of advantages is a result of the interaction of power amongst actors. This statement suggests that mango market chain performance is poor. Collectors are more favored than growers in this inefficient mango market chain. In general, as compared to small-scale farmers, intermediaries had high benefit shares. When the channel has more intermediaries, product prices will be higher and the share of the grower will be lower, which implies that the shorter the channel, the lesser the marketing costs and low-priced the commodity to the end-user.

The results of marketing margin calculations for actors in the mango market chain in the study area revealed that local collectors got the highest marketing margins. This suggests that local collectors in the mango market chain can make a reasonable benefit on their sales if they can minimize operating expenses such as labor costs. The study by Woldu et al. (2015) verified that poorly regulated marketing practices tend to result in marketing margin discrepancies across Ethiopian banana channels. They stated that organizing farmers improve the efficiency of market chains. A large portion of farmers' share in consumer prices goes to local collectors, implying that the involvement of mediators lowers the growers’ benefit shares.

In general, in this study, all market participants were profitable, but small-scale farmers were comparatively disadvantaged by the market because they received the smallest share of the price paid by consumers. As a result, small-scale farmers were comparably disadvantaged in the apple and mango markets, and their market chains did not farewell. This implies that the apple and mango market chains were less effective due to poor vertical/horizontal integration and coordination, as well as insufficient support from institutions in the study areas. Kind of literature such as Mmari (2015) and Kilelu et al. (2017) argued that effective intermediary institutional forms are intended to support combined vertical and horizontal market chain collaboration in order to properly connect farm households into agri-food chains.

## Conclusions and recommendations

This study attempted to characterizing production systems and market performance of apple and mango fruits in northwest Ethiopia using data from a cross-section of small-scale farmers. With this, the study has made substantial contributions to the empirical evidence on fruit crops production systems as well as market performance. Results indicated that there are a significant number of mango and apple farmers in the study areas, with various levels of production and marketing intensity. Unfortunately, farmers have not taken advantage due to poor market pricing for their product and the high expense of post-harvest losses. That is, post-harvest wastage prevents farmers from obtaining anticipated income. It was also concluded that apple and mango trees are intercropped with other crops and planted haphazardly without correct spacing. In consequence, this affects the quality and productivity of apple and mango plantations. The central point drawn from the findings would be that in terms of large sales volumes, a channel connecting mango growers to wholesalers via collectors was more efficient, whereas a channel connecting apple growers to retailers via collectors was more efficient. Similarly, the empirical results from this study confirm that the majority of gross marketing margin goes to traders in both market chains. The key message drawn from the results is that although the production of apples and mango in the study area was profitable, it is clear that farmers did not completely benefit from the production and marketing of apples and mango. As a consequence, farmers have not received fair sales volumes and have been forced to sell their products at low prices. These all deter farmers from producing in large quantities, quality, and also uncertainty (fair failure in the mind of farmers) in the marketing of apples and mangoes.

Based on the findings of this study, the following are potential areas of intervention for the agriculture offices as well as other development practitioners, who are going to support the fruits (apple and mango) production and marketing in the study districts. Apple and mango productivity in the study area is low, implying a higher effort requirement in supporting small-scale producers in terms of training and advice on the agronomic practice of apple and mango production. Train farmers about land preparation, planting, weed control, fruit management, compost preparation, soil moisture control, and post-harvest handling to boost apple and mango production and productivity is essential. Besides, the greater price disparity between farmers and intermediaries/traders indicates that farmers received little assistance. Therefore, establishment of fruits marketing cooperative is required in the study areas to strengthen farmers bargaining power. In line with this, empowering of small-scale farmers to be engaged in vertical and/or horizontal integration is quite important for the efficient use of apple and mango farming and maximizing household’s income. Furthermore, encouragement of farmers to use calibrated weight balances when selling apple and mango produce is required. To reduce cheating on weight balance, development agents should advise farmers on how to properly implement these measurement tools for apple and mango produce marketing. More importantly, agricultural research institutes such as *Pawe* and *Adet* should play a key role in identifying disease-resistant and high-yielding cultivars with the goal of enhancing apple and mango production.

## Data Availability

It is possible to ask the corresponding author for the data.
